# Novel mouthpiece for reducing the gag reflex during esophagogastroduodenoscopy

**DOI:** 10.1111/den.13511

**Published:** 2019-10-02

**Authors:** Kazunori Fujiwara, Kazuya Matsumoto, Naoki Ueda, Masaru Ueki, Takahiro Fukuhara, Yuichiro Ikebuchi, Hajime Isomoto, Hiromi Takeuchi

**Affiliations:** ^1^ Department of Otolaryngology, Head and Neck Surgery Faculty of Medicine Tottori University Tottori Japan; ^2^ Division of Medicine and Clinical Science Department of Multidisciplinary Internal Medicine Faculty of Medicine Tottori University Tottori Japan; ^3^ Advanced Medicine, Innovation and Clinical Research Center Tottori University Hospital Tottori Japan; ^4^ Irisawa Medical Clinic Matsue Japan; ^5^ Department of Gastroenterology Yasugi Municipal Hospital Shimane Japan

**Keywords:** cephalometry, esophagogastroduodenoscopy, gag reflex, mouthpiece, pharynx

## Abstract

**Background and Aim:**

Discomfort associated with the gag reflex during transoral endoscopy can be troublesome. To overcome this problem during esophagogastroduodenoscopy (EGD), we recently developed a novel mouthpiece. The aim of the present study was to compare acceptance and tolerability of transoral EGD with conventional and new mouthpieces in unsedated patients and analyze the effects of the new mouthpiece.

**Methods:**

This study consisted of two phases of cephalometric and EGD examinations to analyze the effects of the new mouthpiece. Cephalometry was carried out in six subjects to evaluate differences in the size of the pharynx (anteroposterior diameter of the oropharynx and longitudinal diameter of the oral cavity) when subjects held the conventional mouthpiece (MAJ674) or the new mouthpiece in their mouths. EGD was done in 10 subjects using the conventional or new mouthpiece to evaluate the number of times the gag reflex occurred, examinee discomfort, and endoscope operability during EGD using a visual analogue scale (VAS).

**Results:**

Anteroposterior diameter of the oropharynx and longitudinal diameter of the oral cavity were significantly larger with the new mouthpiece than with the conventional mouthpiece (oropharynx: *P *= 0.03; oral cavity: *P *=* *0.03). With the new mouthpiece during EGD, subjects had significantly fewer instances of the gag reflex (*P *=* *0.01); VAS score for discomfort was significantly lower (*P *<* *0.01) and score for endoscope operability was significantly higher (*P *=* *0.04).

**Conclusion:**

The new mouthpiece we developed reduced the gag reflex during EGD by extending the pharynx, thus decreasing examinee discomfort and increasing endoscopic operability.

## Introduction

Esophagogastroduodenoscopy (EGD) involves passing an endoscope down the throat and along the length of the esophagus. EGD is carried out to diagnose and treat certain disorders of the upper gastrointestinal tract and for screening purposes.

Sedation for esophagogastroduodenoscopy is routinely used in North America, but it is not common in Japan.^1^, ^2^ More than 75% of EGD in European countries and 32.6% of EGD in Japan were carried out without sedation.^3^, ^4^ In contrast, surveillance studies from the USA have reported that more than 98% of EGD and colonoscopies are carried out with endoscopic sedation.^5^, ^6^ However, EGD under sedation has several problems such as longer examination time, higher cost, and intensive care. Thus, EGD without sedation is considered useful, even though there are several problems associated with transoral EGD such as the gag reflex, choking, and saliva aspiration.

To reduce the aspiration of saliva, a new continuous suction mouthpiece was developed. The new continuous suction mouthpiece reduced the number of episodes associated with salivary flow.^7^ To reduce the gag reflex and choking, transnasal EGD without sedation using a small‐caliber endoscope has been increasingly used in small health institutions and private clinics in Japan. However, transnasal EGD is associated with issues such as nasal pain, epistaxis, longer examination time, and lower resolution.^1^, ^8^, ^9^

In recent years, endoscopic procedures such as sclerotherapy for varices and stomach ulcers, foreign body removal, balloon dilation, percutaneous endoscopic gastrostomy tube placement, and endoscopic submucosal dissection for the esophagus have been developed. These treatments require a large‐caliber transoral endoscope and transoral EGD remains an important procedure.

However, an excessive gag reflex during transoral EGD may make observation difficult. Thus, to increase the precision of the examination, distress associated with transoral EGD without sedation such as the gag reflex, choking, and saliva aspiration should be minimized. To solve the problem of the gag reflex, we recently developed a novel mouthpiece named the gagless mouthpiece. The aim of the present study was to compare the acceptance and tolerability of transoral EGD with the conventional mouthpiece versus the new gagless mouthpiece in unsedated patients and analyze the effect of the new mouthpiece. This study was approved by the ethics committee of our institution. Informed consent was obtained from each participant.

## Methods

### Materials

**Figure 1:**
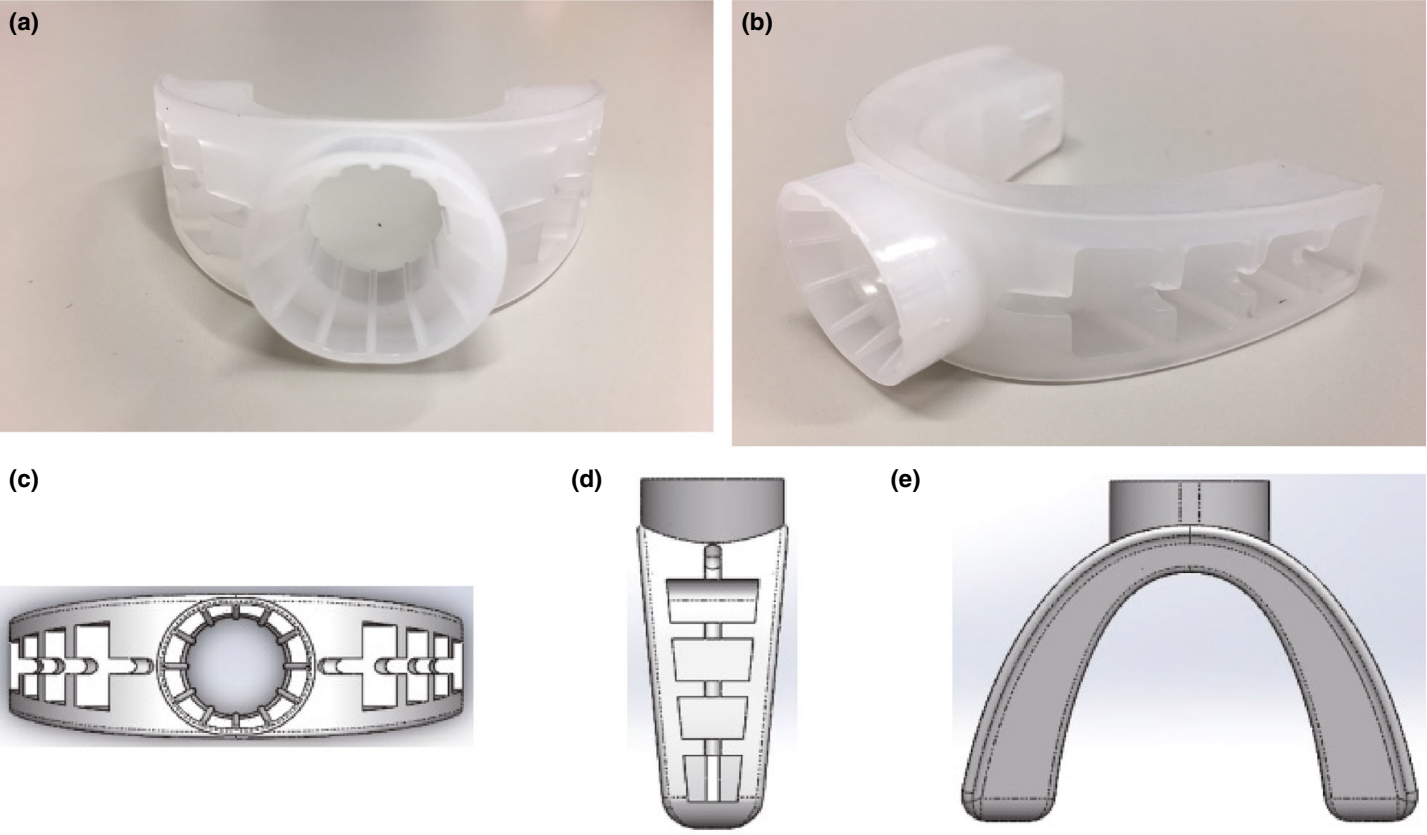
Photo of the new gagless mouthpiece. Front view (a), actual oblique view (b), front view with 3D‐CAD (c), lateral view with 3D‐CAD (d), and superior view with 3D‐CAD (e). CAD, computer‐aided design.

The new gagless mouthpiece for EGD (Fig. 1) is made of elastomer produced by INABA RUBBER (Osaka, Japan) with pharmaceutical approval (general medical device number, 70951000). This mouthpiece was U‐shaped along the entire dental arch. As this mouthpiece is made of a soft material with a cavity located on the lateral side, examinees can hold it comfortably between their teeth. Chewing on it with the back teeth can create occlusal stability. The insertion port located in the center of the mouthpiece has a horn aperture with a groove inside, which facilitates endoscope insertion by reducing friction between the port and the endoscope.

### Cephalometric evaluation

**Figure 2:**
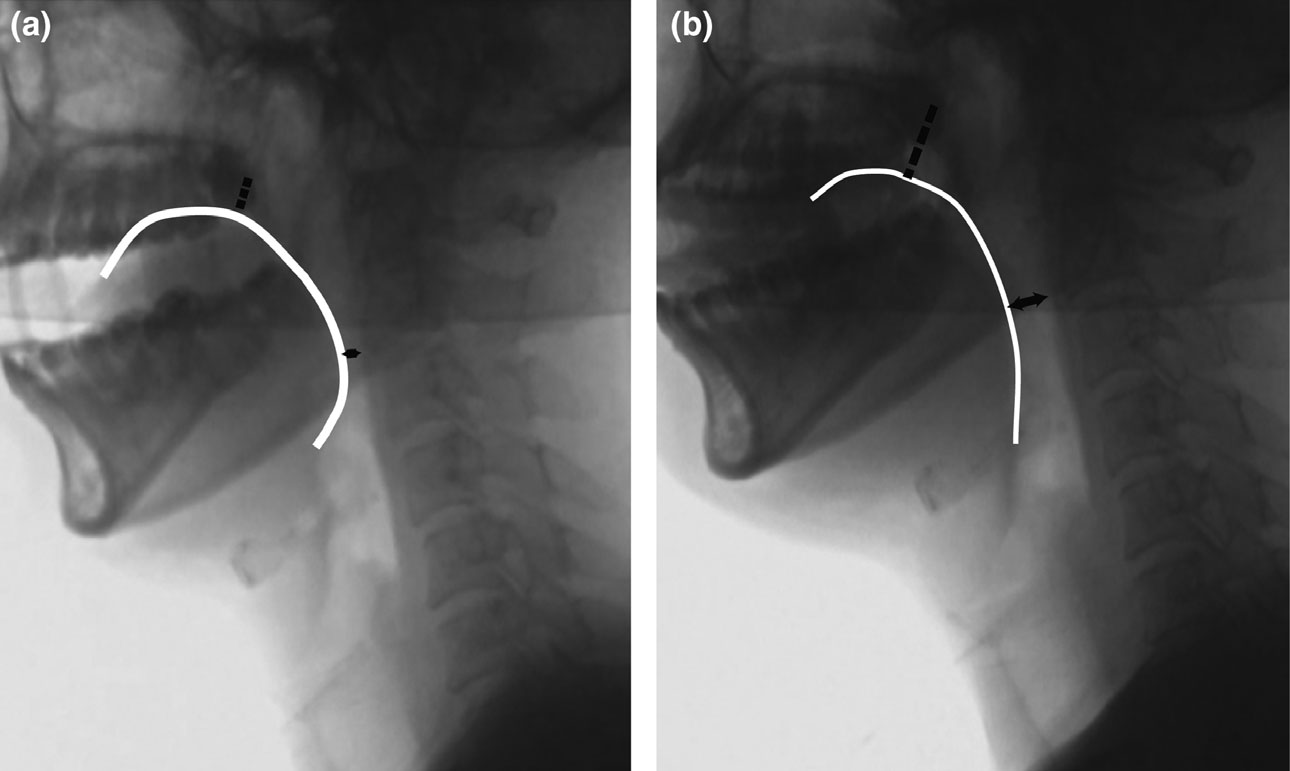
Cephalometry with the conventional versus the new mouthpiece. Anteroposterior diameter of the oropharynx and longitudinal diameter of the oral cavity were measured to determine the size of the pharynx. The edge of the tongue is shown as a white line. The anteroposterior diameter of the oropharynx was defined as the minimum distance between the base of the tongue and the posterior wall (black double‐headed arrow). The longitudinal diameter of the oral cavity was defined as the minimum distance between the dorsum of the tongue and the transition between the soft and hard palates (dotted line). Cephalometry showed that there was less elevation of the base of the tongue with the new mouthpiece compared with the conventional mouthpiece (a: conventional mouthpiece, b: new mouthpiece).

Six healthy volunteers (four males and two females) were recruited (Table 1). In Aug 2016, cephalometry was carried out to evaluate differences in the size of the pharynx when the conventional versus new mouthpiece was held in the mouth. The anteroposterior diameter of the oropharynx and longitudinal diameter of the oral cavity were measured as illustrated in Figure 2. The anteroposterior diameter of the oropharynx was defined as the minimum distance between the base of the tongue and the posterior wall. The longitudinal diameter of the oral cavity was defined as the minimum distance between the dorsum of the tongue and the transition between the soft and hard palates.

**Table 1:** Characteristics of subjects in the cephalometric evaluation

	Age (y)	Height (cm)	Bodyweight (kg)	BMI (kg/m^2^)
1	34	173	75	25.06
2	41	172	60	20.28
3	48	168	60	21.26
4	31	164	55	20.45
5	34	178	73	23.04
6	29	158	52	20.83

BMI, body mass index.

### Esophagogastroduodenoscopy examination evaluation

#### Procedure

Ten healthy volunteers (five males and five females) with no history of EGD were recruited as volunteers and divided into two groups based on the crossover method. Group A included odd‐numbered subjects. Group B included even‐numbered subjects (Table 2). During EGD, group A used the new mouthpiece (gagless) first followed by the conventional mouthpiece (MAJ674; Olympus, Tokyo, Japan). Group B used the conventional mouthpiece first followed by the new mouthpiece.

**Table 2:** Characteristics of subjects in the EGD examination

Case	Gender	Age (y)	Height (cm)	Bodyweight (kg)	BMI (kg/m^2^)	Endoscope	Endoscope diameter (mm)	Group	Endoscopist
1	M	22	173	61	20.38	Q260	9.8	A	1
2	F	23	163	52	19.55	Q260	9.8	B	1
3	F	23	155	45	18.73	HQ290	10.2	A	1
4	F	22	153	42	17.94	H290Z	9.9	B	1
5	M	22	164	56	20.82	H290Z	9.9	A	1
6	M	25	170	52	17.99	Q240Z	10.2	B	2
7	M	52	167	77	27.61	Q260J	9.9	A	2
8	M	42	172	61	20.62	H290Z	9.9	B	2
9	F	33	155	49	20.40	Q240Z	10.2	A	2
10	F	39	165	49	18.00	Q260J	9.9	B	2

All endoscopes were manufactured by Olympus, Tokyo, Japan.

BMI, body mass index; EGD, esophagogastroduodenoscopy.

The use of new mouthpiece is not approved for use in edentulous subjects. Thus, edentulous subjects were not included in the present study. The new mouthpiece comes in just one size, so we used mouthpieces of the same size for all subjects.

From April 2017 to May 2017, procedures for all patients were carried out by two endoscopists in similar ways. The first endoscopist was in charge of EGD for the first half of the subjects and the second endoscopist was in charge of EGD for the second half of the subjects. Both had significant experience (over 10 years) with EGD. Premedication with anticholinergic agents or glucagon was not used. Xylocaine jelly was used in the pharynx before insertion of the endoscope in all volunteers. Sedation was not carried out. During endoscope insertion, the patient was placed on the left side. The endoscopists inserted the endoscope into the proximal stomach. When the esophagus was observed, they used air to inflate the esophagus. The following endoscopes were used for all evaluations: EVIS LUCERA OLYMPUS GIF TYPE Q260, EVIS LUCERA ELITE OLYMPUS CF‐HQ290, EVIS LUCERA ELITE OLYMPUS GIF‐H290Z, EVIS OLYMPUS GIF TYPE Q240Z, and EVIS OLYMPUS GIF TYPE Q260J (Olympus, Tokyo, Japan).

#### Evaluation

Number of gag reflexes was counted from the beginning of endoscope insertion up to the hypopharynx. Data were collected from volunteers and endoscopists. After EGD, volunteers scored their tolerance of endoscope insertion and discomfort during the procedure using the visual analogue scale (VAS) scale. Subjects were asked to rate discomfort by placing a mark on a 100‐mm VAS. The VAS was positioned horizontally with the extremes labeled “no discomfort” and “unbearable discomfort.” In addition, on the VAS scale, endoscopists scored the ease of carrying out EGD (endoscope insertion). Endoscopists were asked to rate endoscope operability by placing a mark on a 100‐mm VAS. The VAS was positioned horizontally with the extremes labeled “unsatisfactory” and “very satisfactory.”

### Statistical analysis

Data are expressed as means ± SE. Number of times the gag reflex occurred, discomfort score, ease of insertion score, and size of the pharynx were compared between the conventional and the gagless mouthpiece groups using the Wilcoxon signed‐rank test in Prism (GraphPad Software, San Diego, CA, USA).

## Results

### Cephalometric evaluation

**Figure 3:**
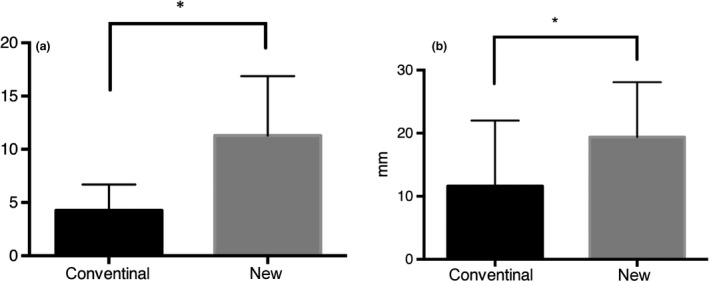
Cephalometric analysis with the conventional versus the new mouthpiece. Anteroposterior diameter of the oropharynx was larger with the new mouthpiece than with the conventional mouthpiece (11.3 ± 2.3 *vs* 4.3 ± 1.0, *P =* *0.03) (a). Longitudinal diameter of the oral cavity was larger with the new mouthpiece than with the conventional mouthpiece (19.4 ± 3.6 *vs* 11.6 ± 14.3, *P =* *0.03) (b).

Comparison of cephalometric parameters between the new and the conventional mouthpiece groups is shown in Figure 3. The anteroposterior diameter of the oropharynx was larger with the new mouthpiece than with the conventional mouthpiece (11.3 ± 2.3 mm *vs* 4.3 ± 1.0 mm, *P *=* *0.03). The longitudinal diameter of the oral cavity was larger with the new mouthpiece than with the conventional mouthpiece (19.4 ± 3.6 mm *vs* 11.6 ± 14.3 mm, *P *=* *0.03). Cephalometry demonstrated less elevation of the base of the tongue with the new mouthpiece compared with the conventional mouthpiece (Fig. 2).

### Acceptance and tolerability of the EGD examination

**Figure 4:**
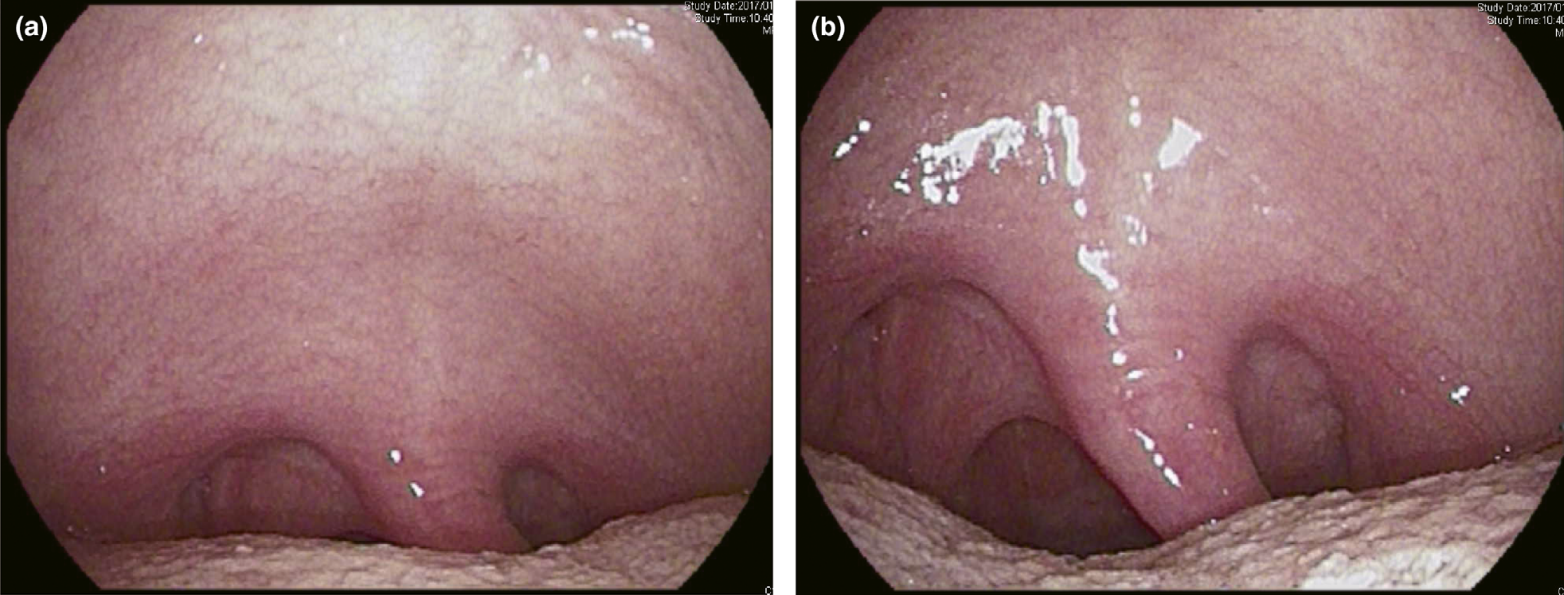
Oropharyngeal findings with the transoral endoscope. Endoscopy demonstrated that the base of the tongue had dropped down and the faucial isthmus became wider with the new mouthpiece (a: conventional mouthpiece; b: new mouthpiece).

**Figure 5:**
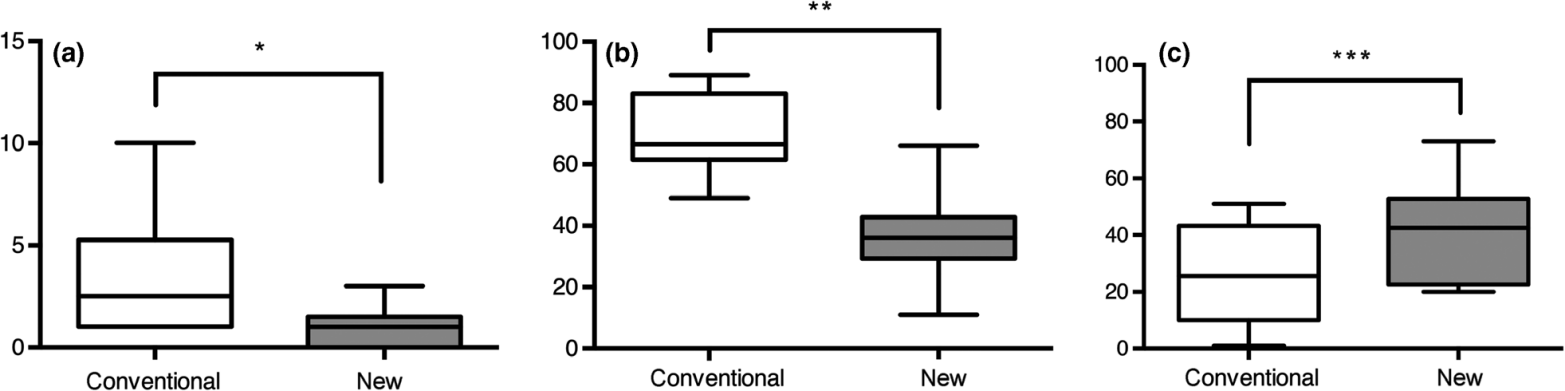
Number of gag reflexes and visual analogue scale (VAS) score by mouthpiece type. (a) Subjects had significantly fewer instances of the gag reflex during esophagogastroduodenoscopy (EGD) with the new mouthpiece than with the conventional mouthpiece (1.1 ± 0.3 *vs* 3.7 ± 0.9, *P =* *0.01). (b) VAS score for discomfort during EGD with the new mouthpiece was significantly lower than with the conventional mouthpiece (36.5 ± 4.2 *vs* 70.1 ± 4.1, **P <* *0.01). (c) VAS score for operability of the endoscope during EGD with the new mouthpiece was significantly higher than with the conventional mouthpiece (41.0 ± 5.3 *vs* 26.8 ± 5.1, ***P =* *0.04).

Esophagogastroduodenoscopy could be completed in all subjects. No adverse events occurred during or after EGD. None of the subjects had abnormal findings. Endoscopic findings included lowering of the base of the tongue and widening of the faucial isthmus with the new mouthpiece (Fig. 4). Procedure time was 47.7 ± 3.8 s in the conventional mouthpiece group and 44.0 ± 4.6 s in the new mouthpiece group. During EGD with the new mouthpiece, there were significantly fewer instances of the gag reflex than with the conventional mouthpiece (1.1 ± 0.3 *vs* 3.7 ± 0.9, *P *=* *0.01). VAS score for discomfort with the new mouthpiece was significantly lower than with the conventional mouthpiece (36.5 ± 4.2 *vs* 70.1 ± 4.1, *P *<* *0.01). VAS score for ease of endoscope insertion with the new mouthpiece was significantly higher than with the conventional mouthpiece (41.0 ± 5.3 *vs* 26.8 ± 5.1, *P *=* *0.04; Fig. 5).

## Discussion

The present study showed that the gag reflex occurred less often during EGD with the new mouthpiece compared with the conventional mouthpiece. Based on VAS scores, there was less discomfort and better endoscope operability during EGD with the new mouthpiece than with the conventional mouthpiece. Cephalometry showed that the new mouthpiece extended the pharynx.

Discomfort associated with the gag reflex during transoral endoscopy can be quite troublesome. It is the primary cause of a stagnant consultation rate. To reduce problems associated with the gag reflex, small‐caliber transnasal EGD has been developed.^9^, ^10^ The mechanical reasons why transnasal EGD is less stressful than transoral EGD remain unclear.^11^ Severe discomfort during transnasal EGD, including nasal pain, has been reported.^1^, ^10^, ^12^ In contrast, transoral endoscopy has higher resolution and magnification than transnasal endoscopy. Thus, transoral EGD has the advantage in terms of detecting lesions. However, transoral insertion may irritate the uvula, palatine arch, and posterior tongue, which may stimulate the sympathetic nervous system.^13^ Thus, preventing the endoscope from touching the pharynx might contribute to solving problems such as transoral EGD discomfort. To prevent the endoscope from touching the pharynx, expansion of the pharynx is required.

Inoue and Yamada reported that the activity of the styloglossus muscle, known as a tongue‐retracting muscle, occurs from the late mouth‐opening phase to the early mouth‐closing phase. Relaxation of the styloglossus muscle occurs from the late mouth‐closing phase to the early mouth‐opening phase.^14^ During the slow closing phase, the tongue was located lower and more posteriorly.^15^, ^16^ The most common conventional mouthpiece model is shaped like a roll in order to hold the incisors. The conventional mouthpiece, which is fixed with clenched front teeth, results in occlusal imbalance. Thus, with a continuous fast‐closing phase, styloglossus muscle activation results in elevation of the tongue and narrowing of the oral cavity and pharynx.

As a result of styloglossus activity, arrangement of the mouthpiece structure affects the morphological changes of the pharynx, which led us to develop a new mouthpiece. The new mouthpiece is U‐shaped along the entire dental arch, which was mostly fixed with the back teeth, resulting in stable occlusion. Thus, with a continuous slow mouth‐closing phase, styloglossus muscle inactivation results in dropping of the tongue and extension of the oral cavity and pharynx. In the present study, cephalometry showed that the oral and pharyngeal spaces were wider with the new mouthpiece than with the conventional mouthpiece. This study is the first to objectively evaluate the extent of the pharynx with cephalometry.

Transoral insertion requires a sharp angle in the pharynx. This might thrust the scope toward the posterior pharyngeal wall more intensely than during transnasal insertion, which may stimulate the sympathetic nervous system. However, in the present study, subjects had fewer gag reflexes during transoral EGD with the new mouthpiece. Expanding the pharynx might have resulted in less contact between the endoscope and the pharynx, thus reducing the gag reflex. As demonstrated above, extension of the pharynx by the new mouthpiece was achieved with clenching of the mouthpiece with the back teeth and a wide pharynx, which decreased contact between the endoscope and the pharynx and resulted in a decreased gag reflex and less pain. This is the first mouthpiece that extends the pharynx.

Recently, to reduce the gag reflex during EGD, other new mouthpieces have been developed. ENDO LEADER (Top Corp., Tokyo, Japan) is an EGD mouthpiece with an upward sloping soft tube that prevents the endoscope from touching the base of the tongue and thus decreases the gag reflex. However, ENDO LEADER does not extend the pharynx; it just guides the endoscope. Our mouthpiece extended the pharynx physiologically, which is the first to have this effect in decreasing the gag reflex.

The lateral and anterior walls of the oropharynx, postcricoid area, and posterior wall of the hypopharynx are difficult to observe using transoral endoscopy.^17^ Hamada *et al*. reported that it is important to observe the oral cavity and oropharynx without a mouthpiece.^17^ In contrast, Tsuboi *et al*. reported that 73.2% of patients received a complete pharyngeal examination during transoral EGD, compared with 98.9% of patients during transnasal EGD. However, transoral EGD provides better image quality. Thus, the study of Tsuboi *et al*. did not determine whether transnasal EGD can detect pharyngeal lesions more effectively than transoral EGD.^18^ In the present study, the new mouthpiece improved EGD operability. In transoral EGD, pharyngeal extension from our mouthpiece might help facilitate pharyngeal cancer detection.

The present study had several limitations. This study was not blinded, included a small sample size, and did not include edentulous subjects. This study did not compare differences in endoscope diameter, mouthpiece size, and sedation. The relationship between pharynx size and EGD acceptance was not evaluated because the subjects who underwent cephalometry were different from the subjects who underwent EGD. Further study is needed.

## Conclusion

Our newly developed mouthpiece reduced the gag reflex during EGD by extending the pharynx, thus decreasing examinee discomfort and increasing endoscope operability.

## Acknowledgment

This study was funded by Medical Equipment Development Business Assistance from Tottori .

## Conflicts of Interest

Authors declare no conflicts of interest for this article.
